# Orbital Burkitt lymphoma: a case presentation

**DOI:** 10.1186/1471-2415-14-109

**Published:** 2014-09-08

**Authors:** Chichua Alexander, Chichua George, Jikurashvili Tinatin, Saginashvili Maia, Mchedlishvili Maia, Pavlidis Mitrofanis

**Affiliations:** Chichua Eye Clinic Mzera, Medical-Research Center Tbilisi Georgia, Tsinandali Str 9, 0144 Tiflis, Georgia; V.Ivrieli Clinic “ENMEDIC” Department of clinical pathology, Tbilisi, Georgia; Augencentrum Köln, Josef Str 14, 51143 Cologne, Germany

**Keywords:** Burkitt lymphoma, Orbitotomy, Chemotherapy BFM-90

## Abstract

**Background:**

Highly aggressive Burkitt lymphoma (BL) with rapidly progressive painful proptosis of the right eye is rarely encountered.

**Case presentation:**

A 31-year-old Caucasian female presented with rapidly progressive painful proptosis of the right eye and poor visual acuity. Diagnostic Computer tomography, magnetic resonance imagining and angiography, identified an oval-shaped mass on the medial rectus of the right orbit that caused dislocation of eyeball, for which she underwent medial orbitotomy. The mass was histologically confirmed as BL, and postoperative aggressive chemotherapy (BFM-90) was initiated. BFM-90 reduced the recurrence of strabismus, diplopia, and proptosis, but did not correct deficits in the best corrected visual acuity.

**Conclusion:**

In patients presenting with painful proptosis and vision loss, a diagnosis of BL should be considered. In addition, because of the rapid progression of BL, and considering that it responds well to treatment, a diagnostic work-up including a tissue biopsy should be initiated immediately if BL is suspected.

## Background

Burkitt lymphoma (BL) is a highly aggressive, small, non-cleaved B-cell non-Hodgkin lymphoma that was first described in 1958 as a mandibular malignancy in African children, and according to the World Health Organisation (WHO), is classified as a mature B-cell neoplasm [1, 2]. BL tumours can arise due to disruptions in the *c-myc* gene caused by translocations between chromosomes 8 and 14. In addition, the presence of antibodies against Epstein-Barr antigens has been associated with the development of African-type BL [3]. In the sporadic form of the disease that occurs worldwide in non-endemic areas, mostly in developed countries, patients present with an abdominal mass that frequently involves the ileocecum in the bowel [4], whereas ocular or orbital involvement is rare [5]. According to a 2012 analysis of the Georgian population, BL is uncommon, and only 47 cases of nodular and 181 cases of diffuse non-Hodgkin lymphoma have been registered [6]. Therefore, because of its low incidence in the Georgian population, we decided to present an atypical case of BL.

## Case presentation

A 31-year-old Caucasian Georgian woman visited our clinic complaining of rapidly progressive painful proptosis of the right eye that had begun two weeks earlier. Approximately two months earlier, she had received antibiotics for sinusitis.

**Figure 1:**
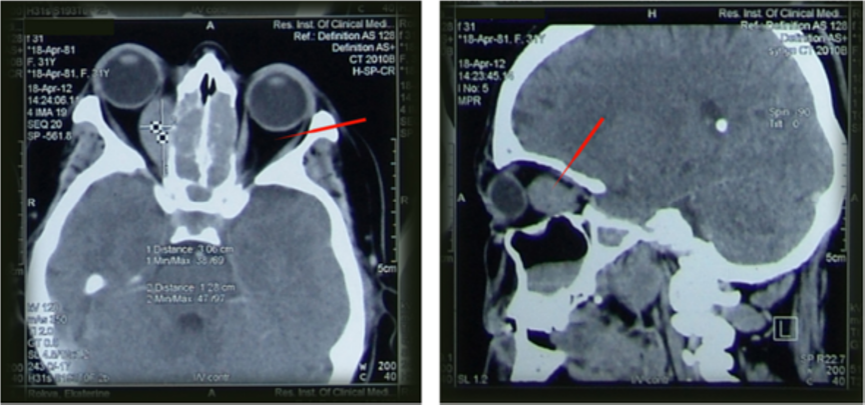
**A computed tomography scan of the paranasal sinuses shows heterogeneous structural opacification of the ethmoidal, sphenoidal and frontal sinuses, mainly at the soft tissue; low-density opacification of the maxillary sinuses, mainly of the viscous fluid; and oval-shaped soft neoplastic tissue mass, 3.1 × 1.3 mm with sharp margins on the medial rectus of the right orbit that caused anteriotemporal dislocation of the eyeball (red arrow).**

**Figure 2:**
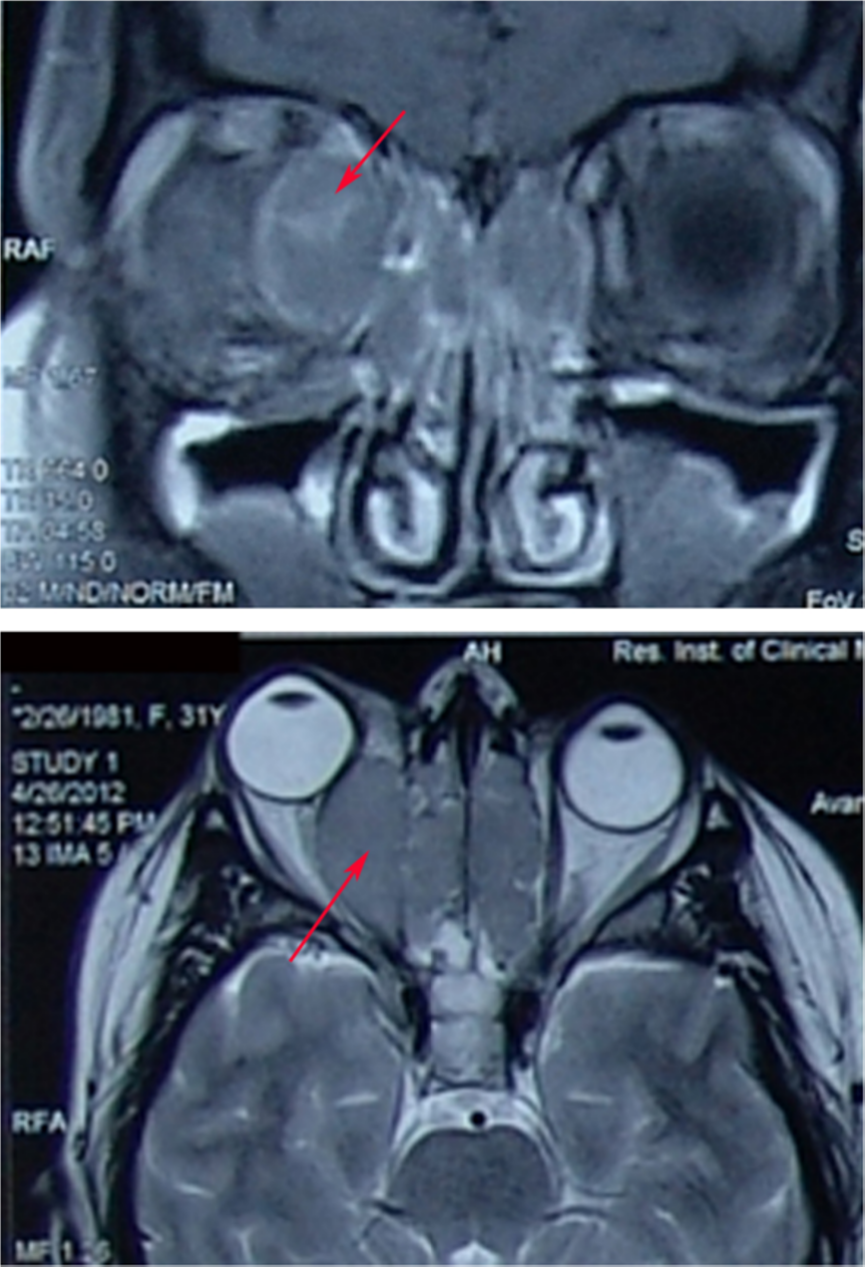
**Brain magnetic resonance imaging and angiography of the cranial vessels using intravenous contrast agent shows right-sided proptosis and an oval structure with highly dispersed protein content (red arrow).**

At presentation, her best corrected visual acuity (BCVA) for both eyes was 1.0 decimal 20/20. Slit-lamp and dilated fundoscopic examinations were normal, and there were no significant changes in the anterior and posterior segments of the eyes. There was no evidence of chorioretinal folds, optic nerve oedema, or pallor. Hertel exophthalmometry readings revealed a 5-mm protrusion of the right eye, but no restriction of eyeball movement or binocular diplopia. Her complete blood cell count was within the normal range. A computed tomography scan of the paranasal sinuses showed heterogeneous structural opacification of the ethmoidal, sphenoidal and frontal sinuses, mainly of the soft tissue; low-density opacification of the maxillary sinuses, mainly of the viscous fluid; and oval-shaped soft neoplastic tissue of 3.1 × 1.3 mm with sharp margins on the medial rectus of the right orbit that had caused anteriotemporal dislocation of the eyeball (Figure 1). Brain magnetic resonance imaging and angiography of the cranial vessels with an intravenous contrast agent identified right-sided proptosis and oval-shaped structure with highly dispersed protein content (Figure 2). There was no intracranial structural pathology, and a diagnosis of a retrobulbar abscess with inflammation of the paranasal sinuses was made.

**Figure 3:**
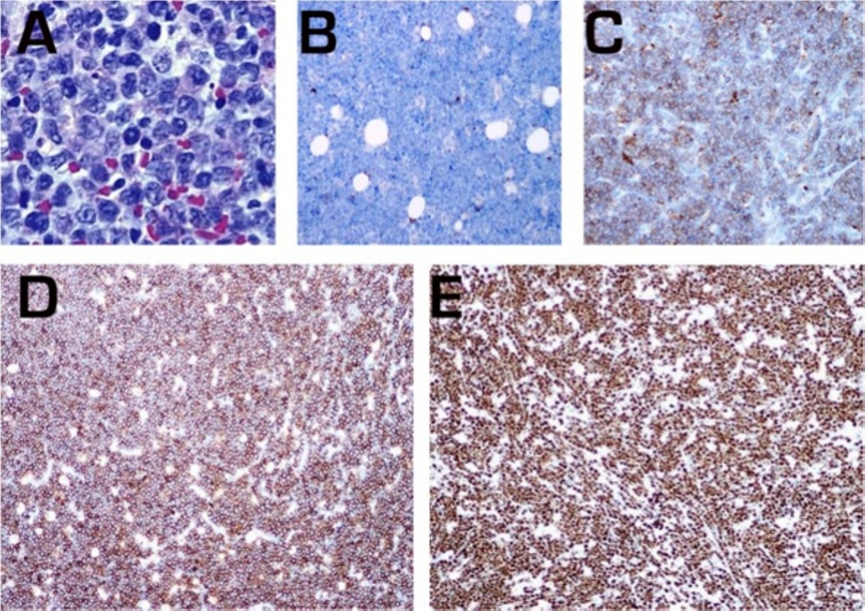
**Immunohistological analysis confirmed the excised tumour as BL. A)** Characteristic “starry sky” appearance of BL. **B)** Staining with BCL2. **C)** Staining with CD10. **D)** Staining with CD20. **E)** Staining with ki-67.

Right-sided medial orbitotomy was conducted under general anaesthesia, and a 1 × 3-mm soft tumour was excised. Histomorphological analysis of the excised tissue revealed a “starry-sky” appearance characteristic of BL. Immunohistochemical evaluation of the tissue against a panel of markers, including CD20, CD19, CD79a, CD3, CD30, CD15, epithelial membrane antigen, anaplastic lymphoma kinase, and the ki-67 proliferation marker confirmed the diagnosis of idiopathic BL (Figure 3).

During the postoperative period, the patient developed oedema of the orbital tissue and eyelids, proptosis, external strabismus, and problems with eye movement and diplopia. She was referred to the Oncohematological Department. Her complete blood cell count was within the normal range, and there were no changes on a bone marrow biopsy. Cytological analysis revealed a negligible increase in lymphocyte count in the cerebrospinal fluid. Aggressive BFM-90 chemotherapy with methotrexate, ifosfamide, and dexamethasone was initiated and was administered every 20 days for six cycles.

Three months after the initiation of chemotherapy, strabismus and diplopia had resolved, and proptosis markedly decreased, although there was no change in her BCVA. A CT scan showed bilateral low-density opacification of the maxillary, frontal, and sphenoid sinuses and oedematous enlargement of the right medial rectus, but no opacification of the retrobulbar space.

## Conclusions

BL is a rare form of non-Hodgkin lymphoma that is detected worldwide. BL is an aggressive, rapidly proliferating cancer, in which tumours can double in size within <24 hours [5].According to the WHO classification, there are three types of BL. Endemic (African) BL primarily affects African children aged 4–7, is twice as common in boys compared with girls, and mainly involves tumours of the jaw or other facial bones. However, it can also affect the gastrointestinal tract, ovaries, and breasts and metastasise to the central nervous system, causing nerve damage, weakness, and paralysis. Sporadic (non-African) BL accounts for 1–2% of adult lymphoma cases worldwide. In the US and Western Europe, 40% of paediatric lymphoma cases are reported as sporadic BL. Immunodeficiency-associated BL is the most prevalent form of BL occurring in individuals with HIV/AIDS, accounting for 30–40% of non-Hodgkin lymphoma in these patients and being described as an AIDS-defining disease. It also can occur in people with congenital immune deficiency disorders and patients receiving immunosuppressive drugs after organ transplant.

While each patient is different, the most common symptoms of BL include painless swelling of the lymph nodes in the neck, chest, abdomen, underarm, or groin; fever; sore throat; night sweats; fatigue; and weight loss or decreased appetite.

A previous case report has described BL mimicking an orbital abscess and painful proptosis in a patient with a history of paranasal sinus inflammation. Although rare, the rapid growth of BL within the orbit can lead to optic nerve compression and vision loss, and while the survival prognosis for patients with advanced BL is good at up to 80% [7–9], unrecognised orbital involvement can lead to irreversible vision loss and lifelong morbidity. In conclusion, in patients presenting with painful proptosis and vision loss, clinicians should consider a diagnosis of BL because of its rapidly progressive nature but good response to treatment. If orbital BL is suspected a diagnostic work-up, including a tissue biopsy, should be initiated immediately to facilitate timely treatment.

## Consent

Written informed consent was obtained from the patient for publication of this case report and any accompanying images. A copy of the written consent is available for review by the Editor of this journal.

## Authors’ information

Alexander Chichua MD, PhD, Head of the Chichua Eye Clinic Mzera, Tbilisi, Georgia.

George Chichua MD, PhD. Medical Director of the Chichua Eye Clinic Mzera, Tbilisi Georgia

Maia Saginashvili MD, Ophthalmologist, Chichua Eye Clinic Mzera, Tbilisi, Georgia.

Tinatin Jikurashvili MD, PhD, Ophthalmologist, Chichua Eye Clinic Mzera, Tbilisi, Georgia.

Maia Mchedlishvili MD, PhD, Head of Department of Clinical Pathology, V. Iverieli Clinic “ENMEDIC”, Tbilisi, Georgia.

Mitrofanis Pavlidis MD, Head or retina Department of Augencentrum Köln, Cologne, Germany.

## Abbreviations

BL: Burkitt lymphoma
WHO: World Health Organisation
BCVA: Best corrected visual acuity.
